# A protocol for a multicentre, parallel-group, pragmatic randomised controlled trial to evaluate the NEVERMIND system in preventing and treating depression in patients with severe somatic conditions

**DOI:** 10.1186/s12888-020-02494-3

**Published:** 2020-03-02

**Authors:** Vladimir Carli, Danuta Wasserman, Gergö Hadlaczky, Nuhamin Gebrewold Petros, Sara Carletto, Luca Citi, Sergio Dinis, Claudio Gentili, Sergio Gonzalez-Martinez, Aldo De Leonibus, Björn Meyer, Luca Ostacoli, Manuel Ottaviano, Silvia Ouakinin, Rita Paradiso, Riccardo Poli, Isabel Rocha, Carmen Settanta, Maria Teresa Arredondo Waldmeyer, Gaetano Valenza, Enzo Pasquale Scilingo

**Affiliations:** 1 National Centre for Suicide Research and Prevention of Mental Ill-Health, Karolinska Institutet, Stockholm, Sweden; 2 Department of Neuroscience “Rita Levi Montalcini”, Università degli Studi di Torino, Turin, Italy; 3 School of Computer Science and Electronic Engineering, University of Essex, Colchester, UK; 4 Faculdade de Medicina and CCUL, Universidade de Lisboa, Lisbon, Portugal; 5 General Psychology Department, Università degli Studi di Padova, Padua, Italy; 6 Life Supporting Technologies, Universidad Politécnica de Madrid, Madrid, Spain; 7 Inventya LTD, Daresbury, UK; 8 GAIA AG, Hamburg, Germany; 9 Department of Clinical and Biological Sciences, Università degli studi di Torino, Turin, Italy; 10 Faculdade de Medicina, Universidade de Lisboa, Lisbon, Portugal; 11 Smartex, Pisa, Italy; 12 Research Center “E.Piaggio” and Department of Information Engineering, School of Engineering, University of Pisa, Pisa, Italy

**Keywords:** Depression, RCT, Prevention, Treatment, eHealth, Intervention, Evaluation, Somatic condition, Quality of life, Patients, Nevermind, Horizon 2020

## Abstract

**Background:**

Depressive symptoms are common in individuals suffering from severe somatic conditions. There is a lack of interventions and evidence-based interventions aiming to reduce depressive symptoms in patients with severe somatic conditions. The aim of the NEVERMIND project is to address these issues and provide evidence by testing the NEVERMIND system, designed to reduce and prevent depressive symptoms in comparison to treatment as usual.

**Methods:**

The NEVERMIND study is a parallel-groups, pragmatic randomised controlled trial to assess the effectiveness of the NEVERMIND system in reducing depressive symptoms among individuals with severe somatic conditions. The NEVERMIND system comprises a smart shirt and a user interface, in the form of a mobile application. The system is a real-time decision support system, aiming to predict the severity and onset of depressive symptoms by modelling the well-being condition of patients based on physiological data, body movement, and the recurrence of social interactions. The study includes 330 patients who have a diagnosis of myocardial infarction, breast cancer, prostate cancer, kidney failure, or lower limb amputation. Participants are randomised in blocks of ten to either the NEVERMIND intervention or treatment as usual as the control group. Clinical interviews and structured questionnaires are administered at baseline, at 12 weeks, and 24 weeks to assess whether the NEVERMIND system is superior to treatment as usual. The endpoint of primary interest is Beck Depression Inventory II (BDI-II) at 12 weeks defined as (i) the severity of depressive symptoms as measured by the BDI-II. Secondary outcomes include prevention of the onset of depressive symptoms, changes in quality of life, perceived stigma, and self-efficacy.

**Discussion:**

There is a lack of evidence-based interventions aiming to reduce and prevent depressive symptoms in patients with severe somatic conditions. If the NEVERMIND system is effective, it will provide healthcare systems with a novel and innovative method to attend to depressive symptoms in patients with severe somatic conditions.

**Trial registration:**

DRKS00013391. Registered 23 November 2017.

## Background

Depressive disorders are one of the leading causes of the burden of disease globally. It is the third leading cause of disability worldwide [1] with an estimated one-year prevalence of depression of approximately 3% in the general population [2]. This increases significantly in individuals with severe somatic conditions as there is a high likelihood of developing a comorbid mental illness. The prevalence of depressive symptoms in patients who have suffered acute myocardial infarction (MI) is estimated at 14% [3] while the prevalence in individuals with chronic kidney disease can range between 26 and 39% [4]. Depressive symptoms are defined as symptoms experienced by patients as identified with self-reported or rater-based scales. Depression, as confirmed with a clinical diagnosis and/or identified with ICD codes, in patients with chronic kidney disease has a prevalence that ranges between 21 and 22.8% [4], and between 3 and 28% in cancer patients depending on the cancer type and treatment phase [5]. However, in reality, these estimates are likely to be higher as depression is often under-reported and under-detected [6]. Moreover, these estimates do not account for other comorbidities, which have been shown to increase the risk of depression. A review of the literature on chronic conditions and comorbid depression found 1.7 increased odds of depression in patients with more than one chronic illness compared to patients with only one chronic illness [7].

This added burden of disease increases the strain felt by the patient, impacting the outcome of the primary disease [8] as well as quality of life (QoL) [2], and thereby can result in a reduction in the management of symptoms [6]. A meta-analysis assessing the impact of depression on mortality after MI indicated that patients who develop depression after MI are at a 13% increased risk of subsequent cardiac events and 22% increased risk of all-cause mortality [9]. In addition to the burden on the patient, there is an added burden on the healthcare system. It has been estimated that cancer patients with comorbid depression have a 32% higher healthcare expenditures and are more likely to use emergency care compared to patients who do not have comorbid depression [10].

Research has shown that while this double burden of disease is common in patients with severe somatic conditions, little is done to prevent the progression of depression and other mental disorders. The standard general practitioner’s guidelines for patients diagnosed with any severe somatic condition are to provide information to patients about improving diet, physical activity, and psychological well-being [11] to prevent subsequent illnesses. However, patients often do not adhere to these guidelines [12]. Moreover, severe somatic conditions generally require considerable lifestyle changes and without proper guidance, this can be very difficult for the patient, especially with concurrent depressive symptoms. Currently, in most European Union (EU) healthcare systems, treatment for patients with serious somatic conditions does not incorporate any preventive methods or early diagnosis of the onset of comorbid depressive symptoms.

The NEVERMIND system, which stands for NEurobehavioural predictiVE and peRsonalised Modelling of depressIve symptoms duriNg primary somatic Conditions with ICT-enabled self-management procedures, aims to fill this gap and give patients more control over their mental and physical wellbeing. The NEVERMIND project comprises a research consortium of nine different centres in six countries that include *Spain*, *Sweden*, *Germany*, *Portugal*, *United Kingdom* and the coordination centre in *Italy*. The feasibility of the NEVERMIND project is based on a previously funded EU project, PSYCHE, where a multiparametric monitoring system based on a sensorised shirt and a smartphone was used to gather physiological and behavioural data of patients affected by mood disorders [13, 14]. The NEVERMIND system collects physiological data and psychometric data to assess the risk of developing depressive symptoms. It also contains interactive online cognitive-behavioural therapy (CBT) modules, which are offered to patients based on their symptoms of depression. It is anticipated that the NEVERMIND system will encourage patients to become more self-reliant, engage more in self-care behaviours, use CBT techniques, and increase their confidence in their ability to carry out daily life activities. All these factors should combine and contribute to a new self-image of a patient and aid in reducing depressive symptoms as well as improving the overall quality of life for the patient.

## Objectives

The primary objective is to assess the effectiveness of the NEVERMIND system in reducing depressive symptoms in patients with a somatic condition in comparison to treatment as usual (TAU). Secondary objectives are the effectiveness of the NEVERMIND system in preventing new symptoms of depression, improving quality of life, perceived stigma, and self-efficacy.

## Methods

### Trial design

The study is a multicentre randomised (1:1) control, parallel-group pragmatic trial conducted in Italy and Portugal that will assess the effectiveness of the NEVERMIND system compared to TAU. Patients will be recruited at three clinical centres; Pisa and Turin in Italy and Lisbon in Portugal. It is hypothesised that the NEVERMIND system is superior to TAU. The study will be conducted according to the CONSORT guidelines for randomised controlled parallel, non-pharmacologic trials [15, 16]. The study design is illustrated in Table 1.

**Table 1:**
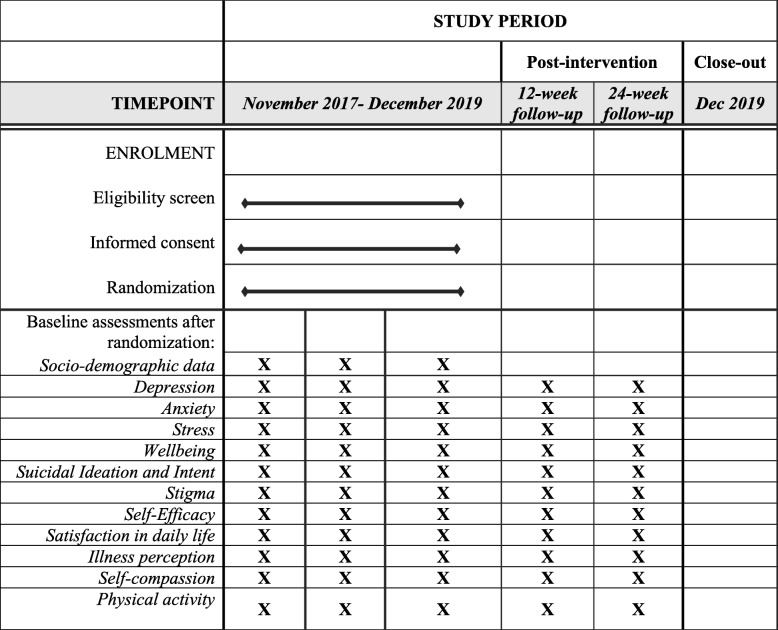
Flow diagram of the study design

Enrolment including eligibility screening and randomisation into either the intervention or control group took place since November 2017, and enrolment will end in December 2019. Baseline assessments were conducted after randomisation and 12-week post-intervention and 24-week post intervention assessments were conducted. The study is planned to end in March 2020.

### Participants

The target sample for each group (intervention and control) is 165 participants, 330 participants in total. The clinical centres selected are those in which the scientific partners are affiliated. Patients from the clinics are referred to the study by their treating physicians, who are also responsible for the patients throughout the trial.

#### Inclusion criteria

Eligible participants are adults aged 18 or older with a diagnosis of one of five following somatic conditions: MI; breast cancer; prostate cancer; kidney failure; and leg amputees. The participants must also be proficient in the language of the country where recruited.

- Patients with myocardial infarction are included if there is a diagnosis of (MI) type I including chest pain for more than 20 min (or equivalent symptoms), acute ECG alterations, and a diagnostic increase of myocardial necrosis biomarkers.
- Breast cancer patients are recruited either at stage II, III or IV.
- Prostate cancer patients are recruited either at stage II, III or IV.
- Kidney failure patients are recruited if they are in a stable clinical condition with chronic kidney failure of stages III, IV, and V as defined by the KDOQI guidelines. This corresponds to an estimated glomerular filtration rate (eGFR) below 60 mL/min/1.73 m^2^ of body-surface area.
- Amputee patients are recruited if they have had a lower limb amputation at any level and are within the 6 months post-surgical intervention.

#### Exclusion criteria

Patients are excluded if they have a past diagnosis of a major psychiatric disorder other than depression. These psychiatric disorders include bipolar disorder, psychosis, and suicidality. Therefore, a diagnosis of depression as a disorder or depressive symptoms is not an exclusion criterion. Moreover, patients are excluded if they have had any recent and active severe suicidality as assessed by the Paykel Suicide Scale [17] or if they have been in treatment with a stable drug therapy for less than two months. Patients are also excluded if they have any cognitive impairment, such as dementia. Moreover, individuals are excluded if they have been involved in other structured psychological treatments involving mindfulness, CBT, other relaxation techniques, or other structured psychotherapy within the three months preceding enrolment. An individual is also ineligible if they have participated or are currently participating in any clinical trial including but not limited to pharmacological trials that might interfere with the study objectives. A lack of capability to participate in study procedures including giving consent will also result in ineligibility. Finally, individuals are ineligible if they demonstrate an inability to use Smartphone technology, especially touch screen interaction and basic maintenance procedures of the devices (e.g. includes charging, switching on and off, reading notifications, making calls, and sending and receiving text messages). Patients’ ability to use Smartphone technology was assessed by asking patients whether they are able to use smartphones to check and send emails.

All patients that meet the inclusion criteria are asked to participate after a comprehensive explanation of the study. Patients provide written consent following the guidelines set by the local ethics committee. Once the patients have consented, they are randomised to either the intervention group or the control group. Subsequently, they are asked to complete the baseline questionnaire. The primary outcome is assessed at 12-weeks after baseline assessment, and a follow-up assessment occurs at 24 -weeks.

### Setting and location

Participants are recruited from three different locations; Turin and Pisa in Italy and Lisbon in Portugal. Kidney disease patients are recruited from the Cisanello University Hospital, University of Pisa, Italy. Breast and prostate cancer patients are recruited from the following centres within the Piedmont Oncological Network, at San Luigi Gonzaga University Hospital, Turin, Italy and Breast Unit-Oncology Department and Urology Department at Città della Salute e della Scienza University Hospital, Turin, Italy. Myocardial infarction patients are recruited from the Cardiology Department at the Santa Maria Hospital, Lisbon. Patients with lower limb amputations are recruited at the Rehabilitation Department at the Santa Maria Hospital, Lisbon. Each of the centres will recruit 75 patients, except for the Rehabilitation department in Santa Maria Hospital which will recruit 30 patients with lower limb amputations. The departments in Turin and Lisbon are the main departments for the region, thus, patients come from all over the region to the clinical centres in Turin and Lisbon.

### Intervention group

The intervention group will receive the NEVERMIND system in addition to TAU, which is described in detail in the next section. The NEVERMIND system is a multifaceted, two-component system that comprises a smart shirt and a user interface, in the form of a mobile application, to put patients at the centre of their health management. The system is a real-time decision support system, aiming to predict the severity and onset of depressive symptoms by modelling the well-being condition of patients based on physiological data, body movement, and the recurrence of social interactions.

The interface monitors the patient’s mental health daily and treats mental health issues as they arise. If a user displays symptoms of depression, they are directed to different levels of treatment in the application. Further targeted advice and care is provided if the user displays symptoms of depression, anxiety, or stress, either through targeted mindfulness or through the online cognitive behavioural therapy programme, Deprexis, provided by Gaia AG [18].

**Fig. 1:**
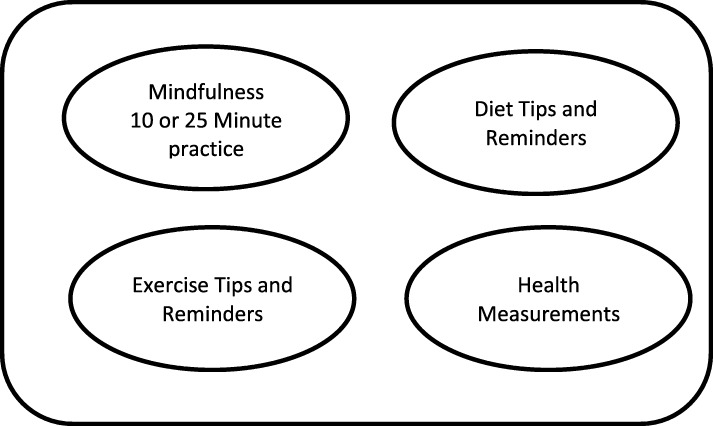
Components freely available to the patients without activation

The user interface also includes an Avatar and a speech monitoring feature to synthetize the system’s messages. In addition, the interface can be personalised to suit the patient’s dietary requirements, exercise recommendations and preferences to provide lifestyle guidance to improve their physical health tailored to their primary somatic condition. This is set up by the treating physician who also enters medical details including weight, blood pressure, and type of condition into the system. Several components are readily available to the patient upon receiving the system (Fig. 1). Lifestyle factors included in the application include diet tips, including recipes, and information about physical activity, including practice exercises. Moreover, there is a section on sleep hygiene and two mindfulness practices per day, either 10 or 25 min long, which can be chosen by the patient.

The smart shirt collects physiological data (biomedical data) including electrocardiogram (ECG), accelerometer data, and respiration data, from which biomedical features are extracted based on signal processing techniques. These features are from three categories: movement dynamics, ECG-heart rate variability, and respiration dynamics.

The patients are encouraged to use the shirt as much as they like but must use it for a minimum of twice a week. The combined data from the interface and the shirt will trigger different responses encouraging patients to perform or alter activities and lifestyle choices to reduce the occurrence and severity of depressive symptoms based on their likelihood of developing depressive symptoms. Based on the general state of the patients, they can be directed to different levels of care as outlined below.

The levels of care (LC) are:

- LC0: Positive feedback for individuals who are managing their mental health and well-being well.
- LC1: Behavioural advice for those showing initial symptoms of depression, anxiety, or stress.
- LC2: Targeted mindfulness if mild or moderate symptoms of depression, anxiety, or stress.
- LC3: Online cognitive behavioural therapy, Deprexis, for those with moderate or severe symptoms of depression, anxiety, or stress.
- LC4: The treatment team will be contacted if patient displays severe symptoms of depression or insomnia for several days in a row.
- LC5: Emergency information and contact of the treatment team if the patient has suicidal intent.

If patients are not responding to a specific treatment and are still reporting depressive symptoms after four weeks, they will be asked if they wish to change to another LC. All patients will receive the NEVERMIND system for 12 weeks. Furthermore, each patient in the intervention group will receive a medical consultation regarding diet, physical activity, sleep, and an introductory session to mindfulness.

To encourage the use of the system and to measure adherence, the system has a compliance monitoring system. If a patient is inactive for three days, that is, not answering the daily questions for three days, a reminder will be sent to the patient. If the user is inactive for seven days, that is not using the system for seven days, the user will receive another reminder and the treatment team will be notified, and they will contact the user to ask the reason for inactivity. If the reason for inactivity is the inability to manage their depressive symptoms, the users will be referred to further treatment.

### Control group

The control group will receive TAU per the standard guidelines at the respective clinics. In the Nephrology ward at the Pisa University Hospital, patients undergo clinical consultations with physicians regularly. The frequency is based on the type of disease and stage of disease of each patient. For patients in pre-dialysis, the visit is scheduled monthly, for patients in dialysis, the visit takes place before every dialysis session, often three times a week. During the visit, an evaluation of mood and anxiety symptoms is performed. If patients report a change in mood or an increase in anxiety level, a visit with a psychiatrist is scheduled within one week. If necessary, medication is prescribed for patients for the relevant psychiatric symptomatology which is compatible with the clinical status of the patient.

In the clinics in Turin, TAU in terms of preventing and treating comorbid mental illness consists of standard clinical management. This includes clinical interviews with the oncologist and eventual referral to the consultation-liaison service, providing assessment, and, if necessary, medications or brief psychological support or both.

The Santa Maria Hospital in Lisbon is a tertiary care unit which has no specific protocol for treating mental health problems. There is a liaison psychiatrist and a liaison psychologist in each of the clinical departments with whom a patient can meet on request. However, treatment for mental ill-health is usually given to patients at the primary care level within the local hospital. Patients receiving psychiatric consultation and medication for depression are retained in the trial.

### Standardisation of intervention administration

Each patient participating in the intervention group receives the standardised NEVERMIND system. All components provided are managed at a central level. The smart shirt and the docking station are distributed by Smartex, and patients are issued the same smartphone with the same user interface. The smart shirt undergoes quality control before it is distributed to the patients. This is done by sending the data through the docking station to the server where the data quality control is checked by looking at the quality of signals received at the docking station. The smartphone is set up so that patients can only use it for the NEVERMIND application. All clinicians and other care providers in both the intervention and control groups are informed to perform TAU only.

### Outcomes

#### Primary outcome

Hypothesis: NEVERMIND will significantly decrease the level of depressive symptoms in the intervention group in comparison to TAU in the control group;

Outcome measure*:* the severity of depressive symptoms as measured by the Beck Depression Inventory II (BDI-II) at T12 (12-weeks post baseline assessment).

#### Secondary outcomes

Secondary outcomes to be measured include general well-being, patient self-efficacy management of primary somatic condition, perceived stigma, prevention of depressive symptoms, and sustainability of the effect of NEVERMIND at 24-weeks post-baseline assessment.

#### Evaluation procedures

Data will be collected at baseline, at 12 and 24 weeks using self-report questionnaires. Moreover, there will be a clinical interview at baseline and at each follow-up. The presence of a psychiatric diagnosis according to DSM-V criteria will also be evaluated during the interview. DSM diagnosis are ascertained by an interview with a psychiatrist at baseline.

The scales that will be administered at baseline and at each follow-up evaluation include the following rater-based and self-report scales:

- the *Beck Depression Inventory II (BDI-II)* which measures depressive symptoms [19]
- the *Hamilton Rating Scale for Depression (HAM-D)* which measures depressive symptoms [20]
- the *Depression, Anxiety and Stress Scale (DASS-21)* which measures depressive, anxiety, and stress symptoms [21]
- the *Hamilton Rating Scale for Anxiety (HAM-A)* which measures anxiety symptoms [22]
- *WHO Well-being Scale (WHO-5)* which evaluates mood, vitality, and general interests [23]
- the *Paykel Suicide Scale (PSS)* which determines suicidal ideation and behaviour [17]
- the *Chronic Illness Anticipated Stigma Scale (CIASS)* which measures perceived stigma [24]
- the *Self-Efficacy for Managing Chronic Disease Scale* which evaluates self-efficacy in managing a chronic disease [25]
- the *Neuro-QoL (Quality of Life in Neurological Disorders) Satisfaction with Social Roles and Activities (8-item)* which assesses satisfaction with daily life when living with a chronic illness [26]
- the *Brief Illness Perception Questionnaire* which assesses illness perception [27]
- the *Self-Compassion Scale (Short Form)* which measures self-compassion [28]
- *the Rapid Assessment of Physical Activity (RAPA)* which assesses levels of physical activity [29]

The following socio-demographic data will be collected at baseline: age, sex, education, marital status, employment, socioeconomic status, living arrangement, and nationality.

#### Sample size

The NEVERMIND system was newly developed and is tested in this study for the first time. Therefore, we could not base the sample size calculations on a previously measured effect size. We estimated the sample size based on a previous study that evaluated the effectiveness of Deprexis in reducing depressive symptoms among patients with somatic disorders [30]. A very similar effect size was also found in a meta-analysis that looked at the effectiveness of Deprexis for depressive symptoms in different settings [18]. The sample size was calculated to detect a similar reduction in depressive symptoms (d = 0.54), where a linear mixed model analysis will be used with a power of 80%, two groups, an intercorrelation coefficient of 0.5 and a significance level of 0.05 (sistas-package [31] and lme4-package [32] for the primary outcome. According to power calculations performed with R [33] for the proposed linear mixed model analysis, 110 patients were estimated. Based on the recruitment criteria, which do not stipulate depressive symptoms as an inclusion criterium, we acknowledge that not all patients will exhibit depressive symptoms at recruitment. Therefore, the sample size was adjusted to account for this. We assume that at least 1/3 of the subjects in the sample of patients with chronic conditions will have depressive symptoms at baseline. In order to reach the desired sample size of 110 patients with depression or depressive symptoms, a sample of 330 subjects who complete the study is required to have adequate power to detect medium effects. A drop-out rate of 20% has been accounted for by adjusting the recruitment process.

#### Recruitment

Patients will be given the opportunity to either participate in this trial or not. However, each clinical centre is within a hospital where a wide spectrum of patients with severe somatic conditions are treated and would give us the ideal platform to recruit participants.

### Randomisation

#### Sequence generation & allocation concealment

Participants are randomly assigned following randomisation procedures to either the intervention group or the control group. Block randomisation is used to ensure an equal number of patients in the intervention and control group at each site. The block randomisation randomizes patients in the control and treatment group in blocks of size ten. This is necessary as each clinical centre treats different somatic conditions, and these should be equally distributed between the two groups. Randomisation is centralized and carried out by an electronic system developed by UPM (Universidad Politécnica de Madrid) in collaboration with KI (Karolinska Institutet). This is done to conceal the randomisation from the researchers involved in enrolment and assessment.

Once the patient has met the eligibility criteria and is enrolled in the study, he/she will complete the baseline assessment. The clinician will then connect to a web page and obtain a randomly generated code to be assigned to the patient. The patient will be assigned to either the control group or the intervention group according to the code.

#### Blinding

Blinding of patients and treating physicians to treatment allocation is not possible. The clinical psychologists who evaluate the patients at baseline and the two follow-up assessments are, however, different from those who randomise and allocate the patients to either the intervention or control group as well as provide the intervention group about the NEVERMIND system. All evaluators are blinded to the group allocation. Any incidence of unblinding is reported to and recorded by the respective centres.

#### Statistical analysis

Intention-to-treat (ITT) analysis will be carried out, which hypothesises the superiority of the NEVERMIND system in reducing depressive symptoms in comparison to TAU. A linear mixed model will be used in order to use all available data of each subject and choose an appropriate covariance structure reflecting the potential dependence due to repeated measures. In order to account for the repeated measurements, the baseline measures will be adjusted for in the model [30, 34]. This increases statistical power, accounts for regression towards the mean, and has been recommended for longitudinal analysis of RCTs [35, 36]. Therefore, the ITT for the primary outcome (BDI-II score at 12-weeks) will be used as recommended for statistical analysis of clinical trials by the European Medicines Agency (EMA; codes CPMP/ ICH/363/96 and CPMP/EWP/2863/99). Group (intervention and control), disease type, and hospital will be included as fixed factors.

Comparison of demographic and clinical characteristics at baseline between subjects of varying primary somatic conditions and clusters as well as between intervention and control group will be examined. In case of imbalances of some characteristics, explorative sub-group analyses will be performed.

If a difference between ITT and per-protocol analysis is observed, more weight will be given to the ITT analysis, which is seen as the primary analysis. If the NEVERMIND system does not show superiority effect over treatment as usual for the ITT analysis, we will look at the extent of usage of the different components of the NEVERMIND system. Additionally, secondary analyses of the primary outcome will include per-protocol evaluations and examine the preventive effect of the NEVERMIND system on the incidence of depression (sensitivity analysis). Analyses of the other secondary outcomes (quality of life, perceived stigma and self-efficacy) will be examined in a similar manner.

Loss to follow-up will be handled through multiple imputations [37] if the missingness is below the recommended rate of missingness and the data structure allows for it as recommended by Jakobsen and colleagues [37]. Otherwise, inverse probability weighting [38] will be utilized. In case of loss to follow-up, observations are only imputed for missing outcomes at 24-weeks (12-weeks after the end of the intervention) by imputing the observation recorded at 12-weeks. No imputation will be performed for the primary outcome at the primary endpoint (T12). Sensitivity analyses will be conducted to test the robustness of the results by conducting complete-case and last observation carried forward analyses [30, 34, 35]. If a loss to follow-up occurs after one follow-up (at 12-weeks), the last observation will be carried out to subsequent follow-ups (from 12-weeks to 24-weeks).

#### Data monitoring

The trial does not have a data monitoring committee (DMC) as the study leaders at each centre oversee each of their respective centres, while the project coordinator manages both the scientific and administrative aspects of NEVERMIND without a need of a DMC. Furthermore, the NEVERMIND centre at KI is responsible for the design and the coordination of the RCT, but each centre is responsible for overseeing data collection at their respective centre. However, there is an external review board that oversees the procedures of the trial. The study leaders will communicate regularly with the other members as well as the project coordinator regarding data collection, and the project coordinator ensures smooth internal and external communications. There are no plans to conduct an interim analysis.

#### Potential risks and harms

The risks associated with using the NEVERMIND application are minimal. If participants exceed a certain threshold in terms of depressive symptoms, anxiety, or suicidal ideation while using the application, they are immediately and automatically referred to a Case Manager (CM), which is a dedicated mental health worker employed at the clinical department where the NEVERMIND system is used. The CM will conduct several evaluations and refer the patient to a healthcare provider if a referral is needed. The CM will periodically contact the patient to monitor the effectiveness of the referral for up to six months.

### Trial status

The trial began enrolment in December 2017. Recruitment will be completed by March 2020. The trial has been registered on 23 November 2017 (DRKS00013391). [https://www.drks.de/drks_web/navigate.do?navigationId=trial.HTML&TRIAL_ID=DRKS00013391].

## Discussion

Despite the high prevalence of depression among patients with severe somatic conditions, there are very few interventions available to treat comorbid depression and none, to our knowledge, to prevent depression in these patients. The present study will provide evidence of whether the NEVERMIND system is an effective intervention in treating depression. If proven effective, the NEVERMIND system will significantly reduce the excess encumbrance posed by comorbid mental ill-health in patients with severe somatic conditions.

Firstly, the burden of disease felt by the patient would decrease, as they will now have the tools to help prevent mental health problems from occurring and will also be able to treat any depressive symptoms if they arise. The NEVERMIND system also provides guidance for lifestyle modifications required by the primary somatic condition, which may not be straightforward. This empowerment helps patients by providing a sense of control over their mental and physical health. Moreover, it can result in a perceived decreased burden for caregivers and the healthcare system. Patients using the NEVERMIND system do not need to rely on their caregivers as much, for they have a guidance system they can use instead of placing the demand on the caregiver. The NEVERMIND system also acts as a support system to primary healthcare, resulting in reduced health care visits as the overall health of the patient will improve.

Furthermore, the NEVERMIND system is a novel and innovative technology that has the ability to influence the use of technology in healthcare, as the system could in the future be adapted and used in many different settings. The system could be expanded to include a range of other conditions that have a high prevalence of depressive symptoms such as other cancers including pancreatic cancer or lung cancer, where the prevalence of depressive symptoms is estimated between 33 and 50% for pancreatic cancer [39] and between 11 and 44% for lung cancer. The system could also be adapted to include other conditions such as Human Immunodeficiency Virus (HIV), where depression prevalence is estimated between 22 and 45% [40]. In addition to using NEVERMIND for other conditions with a high prevalence of depression or depressive symptoms, the central machine learning could be adapted to be used in other contexts, such as in a holistic approach to the treatment of obesity or as a healthy weight management system. There is also the potential to develop the system so it can be available on different platforms such as converting the user interface to a smartwatch application or an application on tablet computers.

### Limitations

This RCT is designed as a pilot study to assess the NEVERMIND system as a proof of concept of its effectiveness, and the methods and study plan have several limitations. First and foremost, patients with and without the same primary conditions are recruited in different centres where the TAU is not consistent. This puts a limitation on the results of the effectiveness of the NEVERMIND system as we do not have a standardized control group. Secondly, there is an implication for cluster confounding. We will adjust the analyses for recruitment centre, but the recruited sample does not allow us to stratify the sample by somatic condition, country or clinic of recruitment. Another limitation was the method used to assess the ability to use Smartphone technology in the intervention group. This was assessed by asking patients if they were able to check and send emails using a smartphone, and not through a validated questionnaire. The inability of some patients to use the NEVERMIND might have an influence on the extent of its effectiveness. Similarly, patients who have been part of a pharmacological trial in the past were excluded with the intention of looking at the full effect of the NEVERMIND system without contamination. However, this might have excessively restricted the inclusion criteria. Thirdly, complete blinding is not achievable in this study. Even if different evaluators have been allocated to different groups of patients, we cannot ensure that incidents of unblinding do not happen. However, all incidents of unblinding are being recorded and will be reported with the results of the study. Lastly, the authors acknowledge that the results of this trial will provide an initial indication about the effectiveness of the NEVERMIND system and that more research will be needed in the future to validate and replicate the results of this study in specific categories of patients. However, if the NEVERMIND system will be effective in reducing depressive symptoms in patients with a severe somatic condition, the results of this study have future implications for a wide range of stakeholders including patients, physicians, and the healthcare system.

## Data Availability

Data sharing is not applicable to this article as no datasets were generated or analysed for developing the protocol. Investigators in the study have access to the current dataset as well as to the final trial dataset. There are no plans for granting public access to the full protocol, participant-level dataset, and statistical code. However, these can be made available upon request.

## Abbreviations

BDI-II: Beck Depression Inventory II
CBT: Cognitive Behavioural Therapy
CIASS: Chronic Illness Anticipated Stigma Scale
CM: Case Manager
DASS-21: Depression, Anxiety and Stress Scale
DMC: Data Monitoring Committee
ECG: Electrocardiography
eGFR: Estimated glomerular filtration rate (eGFR)
EU: European Union
HAM-D: Hamilton Rating Scale for Depression
HIV: Human Immunodeficiency Virus
ITT: Intention to treat
KDOQI: Kidney Disease Outcomes Quality Initiative
KI: Karolinska Institutet
LC: Levels of Care
MI: Myocardial Infarction
MNAR: Missing not at random
PSS: Paykel Rating Scale for Anxiety
QoL: Quality of Life
RAPA: Rapid Assessment of Physical Acitvity
TAU: Treatment as Usual
UPM: Universidad Politécnica de Madri
WHO-5: WHO Well-being Scale
